# Triage, decision-making and follow-up of patients referred to a UK forensic service: validation of the DUNDRUM toolkit

**DOI:** 10.1186/s12888-015-0620-9

**Published:** 2015-10-07

**Authors:** Mark Freestone, Deborah Bull, Roz Brown, Neil Boast, Faye Blazey, Paul Gilluley

**Affiliations:** Violence Prevention Research Unit, Centre for Psychiatry, Wolfson Institute of Preventive Medicine, Barts and The London School of Medicine and Dentistry, Queen Mary, University of London, Garrod Building, London, E1 2AD UK; East London NHS Foundation Trust, John Howard Centre, London, E9 5TD UK; Institute of Psychiatry, King’s College, London, UK

**Keywords:** Forensic, Medium security, Admission, DUNDRUM, Triage

## Abstract

**Background:**

Forensic medium secure services in the UK are a scarce but essential resource providing care for those in the criminal justice system with severe mental disorder. Appropriate allocation of beds to those most in need is essential to ensure efficient use of this resource. To improve decision-making processes in a UK forensic service, an admissions panel utilized the DUNDRUM 1&2 (D1 & D2) triage instruments.

**Methods:**

Demographic, diagnostic and clinical information on a prospective sample of referrals to a UK adult forensic service was gathered (*n =* 195). D1 and D2 measures were scored by a panel of clinical managers considering referral information and clinician opinion in reaching their ratings; those not admitted were also followed up.

**Results:**

Within the sample, D1 ratings were predictive of decisions to admit (AUC = .79) and also differentiated between levels of security (F(4) = 16.54, *p <* .001). Non-admission was not significantly associated with increased risk of offending at follow-up. Items relating to self-harm and institutional behaviour did not show a predictive relationship with the panel decision to admit.

**Conclusions:**

Use of a structured professional judgement tool showing good predictive validity has improved transparency of decisions and appears to be associated with more efficient use of resources, without increased risk to the public.

## Background

Provision of medium secure forensic mental health services in the United Kingdom is an expensive resource, providing services for up to 4,000 patients and costing the UK government an estimated £182,000 per patient [1]. As well as acting as a gateway between the more costly high secure hospitals and the community, medium secure services (MSS) are often the first point of contact for patients awaiting trial on a serious charge or convicted of a violent offence. This includes a minority who are found unfit to plead or not guilty by reason of insanity [2].

For this reason, access to MSS must be determined by the level of need for therapeutic security exhibited by each individual, as recommended by the Tilt Report [3] with an emphasis on placing patients in the least restrictive environment which is compatible with their risk and need. Inappropriate placement can be extremely costly either to services—in the event of an incident or escape, or unnecessary placement in high security—or to the community, in the case of patients denied access to service who subsequently reoffend violently. Despite this, there are significant pressures to ensure that commissioned beds in MSS are filled [4], both from the perspective of demand, i.e., that mentally unwell men and women in prison or on remand are given appropriate care—since the UK Mental Health Act does not apply in prisons and non-consenting patients cannot be required to accept necessary treatments for mental disorder- and also supply, as underperforming services may be at risk of being decommissioned.

However, very often access to services in the UK is determined on the basis of unstructured clinical judgement by a single clinician. While this may be seen as a logical solution—especially if the assessing professional will be responsible for the subsequent care of the patient—it leaves scope for disagreement between professionals about the suitability of the patient for the designated service, which can block or delay transfers and has potential to expose the individual clinician to legal challenge. In addition, even very experienced individual clinicians may have differing thresholds for determining what level of security is required and will weight various forms of evidence differently when arriving at their recommendations.

Managers and commissioners need to be able to demonstrate equality of access to services and that highly specialist services are being apportioned according to the service specifications determined by commissioners. It is therefore imperative that mangers develop robust systems to provide assurance in respect of patient selection, access to treatment and discharge from specialist services.

As a result, several decision-support tools have been developed to help clinicians and managers select appropriately-placed referrals to MSS. These include the Level of Service Inventory-Revised (LSI-R) [5], a 54-item checklist designed to help determine the appropriate level of supervision for offenders; the Medium Secure Risk Assessment Guide MSRAG [6], an eight-item measure of recidivism risk in patients referred to medium security; the Security Needs Assessment Profile [7], a 22-item assessment of the physical, procedural and relational security requirements of mentally disordered patients; and a research checklist developed by J Shaw, J Davies and H Morey [8] considering the appropriateness of placement of patients in secure care according to their security, dependency and treatment profile (SDTP).

However, many of these measures are tailored for a specific population, whether offenders in the community, forensic patients in medium security or high security conditions; none have seen widespread adoption, and many different local systems still remain in place across the UK.

### Measures for assessing need for therapeutic security

A recent development in the assessment of need for therapeutic security has been the initial validation of the Dangerousness Understanding, Recovery And Urgency Manual (DUNDRUM Quartet; [9]), a suite of measures that aid the triaging of potential patients into all levels of therapeutic security, as well as the assessment of treatment completion and readiness for discharge. The DUNDRUM Quartet consists of four assessments of appropriateness of patients for admission to, and retention in, forensic mental health services and has seen increased adoption internationally since its publication [10]. The first assessment considers need for admission to forensic services, and has been validated by the authors [11]; the second considers urgency for admission [12]; and the third and fourth assessments assess, respectively, progress in treatment and recovery and have also received preliminary validation [13]. The checklist is particularly appealing to modern forensic mental health services because of its ability to demarcate different levels of need for therapeutic security, from open to high security, and provide a transparent basis for decision-making.

### East London NHS foundation trust forensic service

The East London NHS Foundation Trust Forensic service comprises two secure hospitals: the John Howard Centre, comprising 126 beds designated ‘medium’ security (16 of which are specialist beds for men with severe personality disorder); and Wolfson House, comprising 80 beds at a level of forensic low security (FLS).

In 2011 the service identified a number of indicators of possible inefficiencies in the admission care pathway for patients. The first was a long waiting list, comprising up to 29 individuals requiring admission to the service, the majority of whom were acutely mentally ill, and many without treatment whilst in prison. Second was a lengthy wait pre-admission for those patients who were placed on waiting list. The scarcity of available beds meant that only the most clinically critical cases could be admitted in a timely way and that other cases, deemed urgent but not critical, often remained *in situ*, sometimes with considerable concern about their welfare.

Thirdly, allocation of resources was in part dependent upon the approach taken by individual assessing clinicians and their capacity to influence allocation decisions taken by managers, there being no commonly agreed or objective method available to clinicians or managers to distinguish the degree of urgency and clinical need of all of those awaiting admission. This lack of a commonly agreed or objective method available impaired the ability of managers to argue for additional resource, and made escalation to commissioners—in cases of urgent need beyond available capacity—difficult to evidence.

A final possible inefficiency lay in the context in which clinical assessments were made, which exposed clinicians to criticism that they were unduly influenced by resource constraints when they did not recommend admission for a particular individual. Further, lack of clarity amongst assessing clinicians about the parameters of the commissioned service and exclusions meant that some cases might be recommended for admission only because a more suitable service was not available.

To address these inefficiencies, the Trust developed a workstream to address all stages of the clinical care pathway in the forensic service with a view to improving discharge rates, critically evaluating the current treatment and security needs of all inpatients and developing a standardised and accountable method for determining how decisions were made to admit patients to the specialist forensic service.

As part of this workstream, the service developed an admissions panel, which would oversee all adult mental health referrals—excluding a specialist PD service—seeking admission to medium or low secures beds, irrespective of whether the assessing clinician recommended admission. By including all cases in this manner it would be possible to look at patterns of referral and of need across the area covered by the service and to effect and demonstrate equality of access. The principle of ‘least restrictive alternative’ could be considered as a matter of routine as part of a peer review process, now involving senior social care staff, with a view to preventing ‘drift’ of patients into forensic services when safe alternatives could be devised. The panel would be able to consider organisational risk, particularly in respect of patients deemed to require high secure services and the standardised professional judgement adopted by the panel would support referrals/appeals in such cases. Patients not accepted would be followed up, to provide assurance that the panel system was not generating risk by influencing access to services.

The method adopted by the panel allowed an objective system for comparing the urgency of individual cases, providing an oversight of all cases awaiting admission and thus reduced pressure on individual clinicians. To enable transparent and systematic decision-making, the DUNDRUM quartet D1 and D2 measures were adopted to assist the panel as Structured Professional Judgement aids.

An important principle in developing the panel system was that it would be clinically led and that a peer review process would be adopted. The information derived from the records of the panel meeting would be systematically recorded to provide an auditable trail of the decision making process and to provide quantifiable information to support performance reports and to drive further service improvement.

### Aims

The main aim of this study was to assess the internal and predictive validity of the DUNDRUM measures D1 and D2 in triaging and prioritising admission of referrals to an inner-city forensic low and medium secure service in the UK serving an adult mental health population. The study addressed five research questions:

i) Does the D1 measure show internal reliability and predictive validity for admission of a UK sample of adult patients with severe mental illness (SMI) to forensic secure services?
ii) Does the D2 measure show internal reliability and association with speed of admission for those admitted to services?
iii) What was the level of agreement between an admissions panel working with the D1 and individual clinical judgement of suitability for forensic services?
iv) For a sample of patients declined admission, did the D1 measure show predictive validity in assessing future violence and/or future admission to forensic services?

## Methods

### Design

We used a prospective panel design to consider the predictive validity of the DUNDRUM-1 (D1) and DUNDRUM-2 (D2) measures when used by a newly-constituted admissions panel in determining service outcomes for patients. The study was organised by a senior researcher within the service in liaison with representatives of the panel.

Initially, patients referred to the service would be allocated to a clinician—a forensic psychiatrist—for assessment of suitability for admission. Data on demography and presenting difficulties were collected by the clinician and recorded in file notes, and the clinician would make an initial recommendation about patient suitability to the panel. This information was then used by the panel to score the D1 measure, which was considered by the panel in making decisions i) whether the patient was suitable for forensic services; and ii) whether to offer admission to the service itself, to decline admission, or to refer elsewhere.

For those offered admission, the D2 measure was then scored by the panel in order to determine urgency of presentation in assigning a bed. Patients were typically assigned a priority for admission based on D2 score, except where operational reasons had to take precedence (e.g. an internal transfer for security reasons would be placed above a new admission). Data about the length of time to admission and length of stay for admitted patients was collected from patient service records by the researcher.

Patients not admitted to the Service were not scored on the D2 measure but were followed up through their clinical teams or RC at 6 and 12 months by an experienced senior administrator to determine eventual outcome (e.g. placement in alternative service; release; discharge or re-referral) and whether the patient had been reconvicted after the decision was made not to admit. Patients were not admitted to the service for a variety of reasons including: not having a treatable mental disorder requiring treatment under the Mental Health Act (*n =* 16, 8 %); being manageable in community services without forensic care (*n =* 40, 21 %); requiring high secure care (*n =* 8, 4 %); requiring other specialist placement not offered by the service (*n =* 2, 1 %); suitable for admission but not admitted for other reasons, e.g. Ministry of Justice non-approval (*n =* 5, 3 %); or another reason such as lack of information, wrong catchment or given a placement elsewhere (*n =* 32, 16 %).

Data from admission panel records were collected by the researcher, who pseudonymised them and entered them into a database for the purposes of analysis. Follow-up data collected by the administrator were sent to the researcher using the pseudonymous identifiers.

### Sample

The main sample comprised 195 referrals to the forensic Service made between June 2011 and June 2013, of whom 161 (83 %) were initial (unique) referrals from the perspective of the study, and 34 (17 %) were re-referrals of patients already considered by the panel. 174 referrals (89 %) were male patients and 21 (11 %) were female.

### Measures

The DUNDRUM-1 and 2 (D1 and D2) measures were used as decision-support aids to the admissions panel in determining whether referred patients should be admitted to either medium secure services, low secure services or forensic psychiatric intensive care unit (PICU); or should be declined admission and/or referred to other services at lower or higher levels of security.

The D1 Triage Security tool is a structured professional judgement measure designed to assist clinicians in the triage of patients referred to forensic services, specifically to ensure they are admitted into the appropriate level of therapeutic security for their needs. It comprises 11 items, which are described together with initial psychometric properties, in greater detail elsewhere [11]. The D1 is a ‘static’ or historical rating and is therefore appropriate when assessing the level of therapeutic security to which a patient should first be admitted. It is not appropriate when assessing readiness to step down to a less secure placement, although the D1 has been shown to be complimentary to the HCR-20 when predicting moves to less secure places [14]. However, the D1 has been shown not to predict conditional discharge from a secure forensic hospital [15]—which would be the role of the DUNDRUM-3 and DUNDRUM-4 measures—and not to correlate with some risk assessment measures [16]. However, the D-1, when combined with the other scales of the DUNDRUM quartet, has been found to meet criteria for an acceptable routine outcome measurement in forensic mental health [10].

The D2 is the second part of the DUNDRUM toolkit comprising 6 items related to the urgency with which patients triaged as needing forensic care should be admitted. The D2 has shown promising internal validity and inter-rater reliability in a previous validation study [12].

### Data analysis

Initially, we used summary statistics to describe the sample in terms of demographic and presenting characteristics and also in terms of their D1 scores at an item-by-item level. Internal consistency of the D1 and D2 scales was assessed using the Cronbach alpha (α) measure; corrected item-total correlations (CITCs) and item-level α were also calculated to identify items that correlated weakly with the overall total. Correlations between the D1 and D2 scales were also calculated for both samples using the Pearson product–moment coefficient.

To assess predictive validity of the D1 measure in both samples, the Area under the Curve (AUC) of Receiver-Operant Characteristic (ROC) curves was calculated for the correct prediction of suitability for services for both the total scale score, and individual scale items. Additionally, a median split analysis was used to calculate positive predictive value (PPV) and negative predictive value (NPV) and percentage correctly classified (PCC) for the D1 measure. Where the performance of ROC curves were compared (e.g. for D1 + D2 against D1 alone), this was performed by comparing values of Somer’s *D* statistic of ordinal association [17] for bivariate pairs of ratings on the two predictor variables [18].

Associations between D2 total score and time taken to admission were calculated initially using correlation then, if a significant finding was suggested, using linear regression, controlling for gender and level of security (as different services may have different waiting times). The strength and significance of the relationship was derived, as well as the R^2^ measure of variance explained by the model.

The majority of data analysis was conducted using IBM SPSS for Windows, version 22.0. Analysis of sensitivity, specificity, optimal cutpoints and PCC values was conducted using STATA SE for Windows version 13.1 (64-bit).

### Ethics

This was an audit of admission panel decision-making that did not make use of patient identifiable data and had service-level ethical approval from the East London NHS Foundation Trust Forensic Directorate. Patients were identified by a pseudonymous number that was retained by a researcher and used to link data obtained at the panel with data obtained at follow-up. Follow-up data was collected by the administrator and sent to the researcher using this link pseudonym.

## Results

### Sample description

Demographic details for the sample are described in detail in Table 1. The majority of referrals were male (*n =* 174, 89.2 %), with the single largest ethnic group being Black or Black British (*n =* 84, 43.1 %) and the most common primary diagnosis was schizophrenia (*n =* 98, 50.3 %). 92 of those referred (48.9 %) were deemed suitable for services, with the majority of those accepted offered beds in medium security (*n =* 74; 37.9 % of all referrals).

**Table 1:** Demographic characteristics of the validation sample

		Referral Sample, *n =* 188	
		*n*	%
Age	[Mean/SD]	34.6	*10.7*
Gender	Male	174	*89.2*
	Female	21	*10.8*
Location at referral	Prison - Secure (Cat A or B)	83	*42.6*
	Prison - Open ( ≤ Cat C)	8	*4.1*
	Hospital - Low secure/open	65	*33.3*
	Hospital - Medium Security	6	*3.1*
	Hospital - High Security	16	*8.2*
	Community	8	*4.1*
Ethnicity	Black or Black British	84	*43.1*
	White or White British	36	*18.5*
	Asian or Asian British	35	*17.9*
	Mixed Race	23	*11.8*
	Other	8	*4.1*
Primary diagnosis	Schizophrenia	98	*50.3*
	Other Psychotic Illness	31	*15.9*
	Personality Disorder	20	*10.3*
	Schizoaffective Disorder	19	*9.7*
	Mood Disorder	16	*8.2*
	Other Psychotic Illness	11	*5.6*
Suitability for services	Suitable	92	*48.9*
	Not suitable	96	*49.2*
	Unsure/Deferred	7	*3.6*
Panel recommendation	Admit - PICU	16	*8.2*
	Admit - Low Secure	17	*8.7*
	Admit - Medium Secure	74	*37.9*
	Refer - High Secure	4	*2.1*
	Do not admit	73	*37.4*
	Deferred	11	*5.6*

Considering the impact of demographics on outcome, ethnicity had no significant effect on the decision of suitability for services (*χ*^2^(5) = 4.84, *p =* .436), nor did age (t(180) = −0.998, *p =* .391), gender (*χ*^2^(1) = 2.31, *p =* .128), primary diagnosis (*χ*^2^(6) = 7.26, *p =* .298), or location at referral (*χ*^2^(7) = 8.4, *p =* .272).

**Fig. 1:**
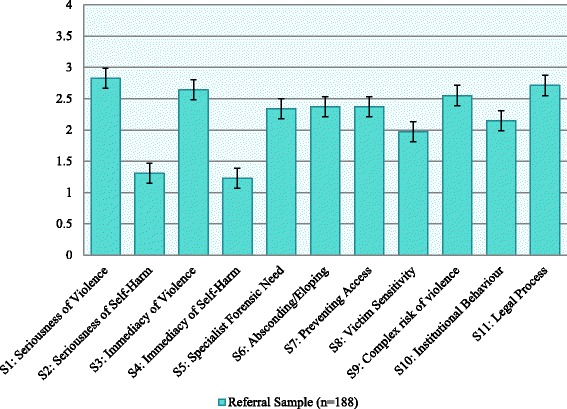
Ratings of D1 items with Standard Error bars

D1 scores were available for 192 observations, and the mean total D1 score for this sample was 24.5 (SD = 6.64) with a range of 9 to 40. D1 total scores followed a normal distribution (Shapiro-Wilk W = 0.99, *p =* .140). Means and standard error bars for individual items are given in Fig. 1 below. 

### Internal consistency

In the SMI sample, the Cronbach alpha (α) statistic for the D1 measure was 0.77, showing a ‘good’ level of internal consistency. Corrected item-total correlations (CITC) for scale items were ≥ 0.3 except for three items: TS1 (History of Serious Self-Harm), CITC = 0.17; TS4 (Immediate Risk of Self-Harm), CITC = 0.28; and TS10 (Institutional Behaviour), CITC = 0.26. Removing these three items from the D1 scale would have resulted in an increased α = 0.85.

### Predictive validity – SMI sample

**Fig. 2:**
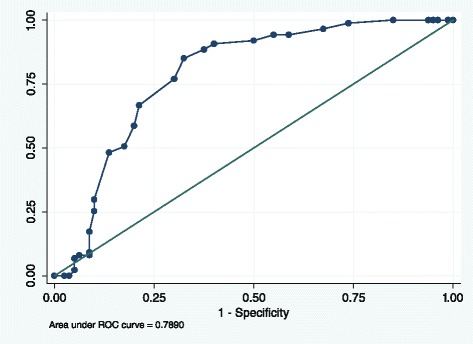
ROC Curve, predictive accuracy of D1 for patients admitted to forensic services (not HSH)

The total D1 scale and sub-scales were used to predict suitability for admission to the service – PICU, low secure forensic or medium secure forensic—using ROC curves; the results are presented in Fig. 2 and Table 2. Individuals referred to or from high security (*n =* 13) were omitted from this analysis so as not to breach the conditions of the AUC algorithm, such that higher scores indicate positive outcome. Those referred for the purposes of step-down from high-security would also have been unrepresentative of the standard clients treated in medium secure forensic services.

**Table 2:** Summary and predictive values of DUNDRUM-1 measure for admission suitability for FLS/MSU

	Referral sample excluding high security (*n =* 175)								
	Mean	SD	Median	AUC	95 % CI	p	Sens	Spec	PCC
S1: Seriousness of violence	2.71	0.92	3	0.70	0.62–0.77	<.001	78.2	61.5	70.0
S2: Seriousness of self-harm	1.29	1.41	1	0.51	0.43–0.59	.799	55.2	47.0	51.2
S3: Immediacy of violence	2.57	1.17	2	0.76	0.69–0.83	<.001	95.4	34.9	65.9
S4: Immediacy of self-harm	1.21	1.39	1	0.53	0.45–0.62	.601	97.7	3.6	51.8
S5: Specialist forensic need	2.23	0.91	2	0.72	0.64–0.79	<.001	94.3	42.2	68.8
S6: Absconding/eloping	2.33	0.88	2	0.69	0.61–0.76	<.001	95.4	22.9	60.0
S7: Preventing access	2.31	0.74	2	0.69	0.61–0.76	<.001	97.7	15.7	57.7
S8: Victim sensitivity	1.90	1.20	2	0.64	0.56–0.72	.001	69.0	59.0	64.1
S9: Complex risk of violence	2.47	1.01	2	0.62	0.54–0.70	.009	94.3	15.9	56.2
S10: Institutional behaviour	2.04	1.06	2	0.57	0.48–0.65	.089	71.3	27.7	50.0
S11: Legal process	2.58	0.91	3	0.76	0.69–0.83	.001	77.0	74.1	75.6
D1 Total score	23.7	6.38	24	0.79	0.72–0.85	<.001	77.0	70.0	73.7
D1 + D2 TOTAL score	38.5	7.62	39	0.74	0.55–0.94	.016	58.7	76.9	61.4

NB: Sens = sensitivity; *spec* specificity; *PCC* Percentage Correctly Classified

When comparing those admitted to MSS, FLS or PICU with those not admitted, the overall AUC achieved for the D1 total score was 0.79 (95 % CI 0.72–0.85), which was significantly better than chance (*p <* .001). At the median value of 24, 72.5 % of cases were correctly classified by the D1 instrument. However, the optimum sensitivity and specificity was achieved at a cutpoint of ≥ 23 points on the TS scale, which gave a sensitivity of 85.1 % and a specificity of 67.5 %, with 76.7 % of cases correctly classified. A full item-by-item ROC analysis of the D1 measure is given in Table 2.

As the table shows, most items on the D1 scale successfully predicted clinical judgement of suitability for services. Three items did not significantly improve over a chance prediction, these were: the two self-harm items (S2 Seriousness of Self-Harm; and S4 – Immediacy of Self-Harm Risk); and the item measuring previous institutional behaviour (S10). Individual items successfully classified between 50 % (S10) and 76 % (S11) of cases as suitable or unsuitable (Table 3).

**Table 3:** DUNDRUM-1 means by panel recommendation

	D1 Score				
Panel recommendation (*n =* 184)	n	Mean	SD	95 % CI (Mean)	
Admit to PICU	16	19.31	4.84	16.92	21.70
Admit to forensic low security	17	19.53	4.84	17.21	21.85
Admit to medium security	74	27.74	3.98	26.83	28.66
Refer to high security	4	33.25	3.10	30.20	36.30
Non-admission	73	22.56	7.54	20.78	24.34

Repeating the ROC analysis following removal of the two low-AUC self-harm items from the D1 total score resulted in an improved AUC of .80 (95 % CI 0.73–0.87), although comparison using Somer’s D test through the lincom function in STATA indicated that this was not a significant improvement (*p =* .173). Removing both the self-harm items and the S10 Institutional Behaviour item reduced the predictive accuracy of the scale (AUC = 0.71, 95 % CI 0.63–0.79), but again this was not significant (*p =* 0.582).

### Sub –group analysis

Of those 105 patients deemed suitable for admission or onward referral, we conducted an ANOVA with post-hoc tests of D1 scores against the panel’s recommendation of the appropriate level of security for the referral (see Table 4). The overall ANOVA was highly significant (F(4) = 16.54, *p <* .001), indicating that the D1 was able to discriminate between therapeutic security profiles of mentally disordered offenders. Contrast ANOVA with Bonferroni correction showed significant differences in D1 scores between those admitted to MSU or HSH and those not offered forensic admission, and also between FLS and HSH; it also showed no difference in D1 scores between those eventually admitted to PICU or FLS. However, the analysis did not show significantly different D1 scores for those admitted to HSH or MSU, and neither did it differentiate between scores for non-admitted and PICU/FLS clients.

**Table 4:** ANOVA comparison of D1 means with bonferroni correction (*n =* 184)

	Mean Differences			
	Refer PICU	Admit FLS	Admit MSU	Refer HSH
Refer PICU				
Admit FLS	0.22			
Admit MSU	8.43***	8.21***		
Refer HSH	13.90***	13.72***	5.51	
Non-admission	3.24**	−3.03	−5.18***	−10.69**

*p < .05, **p < .01, ***p < .001

Additional analysis was then conducted to examine the predictive accuracy of the D1 measure in considering access to varying levels of security. The first analysis was between PICU/FLS (both equivalent to a rating of ‘2’ on the D1 items) and MSU admission (ratings of ‘3’), excluding non-admission and high security cases. This analysis gave an AUC of .91 (95 % CI 0.84–0.98, *p <* .001). A second analysis comparing MSU with HSH cases only also gave a significant AUC of 0.87 (95 % CI 0.72–1.00, *p =* .040).

### Panel and clinician agreement

The level of agreement between the panel and the assessing clinician was calculated by cross-tabulating a binary value of whether the clinician recommended admission or not (to any service) against the panel’s admission variable: see Table 5. The panel and clinician agreed on 66 % of cases (*n =* 107), with clinicians more often recommending admission than the panel assisted by the DUNDRUM (*n =* 51; 31.5 % of cases). Only in four cases (2.5 %) did the panel recommend admission when the clinician had not. This difference in opinions was highly significant (*χ*^2^(1) = 27.3, *p <* .001).

**Table 5:** Consultant vs. SPJ-assisted panel decisions on admission

Consultant recommendation	Panel recommendation		
Admission (yes/no)	No	Yes	Total
No	34 (40.0 %)	4 (5.2 %)	38 (23.5 %)
Yes	51 (60.0 %)	73 (94.8 %)	124 (76.5 %)
Total	85 (52.5 %)	77 (47.5 %)	162 (100 %)

### Admission speed and D-2

When we analysed the relationship between D2 score and time to admission, we found a strong negative correlation, but this was not significant (r = −.33, *p =* .204). Combining the D1 and D2 scores did not improve AUCs (see Table 2).

### Follow-up of patients

**Fig. 3:**
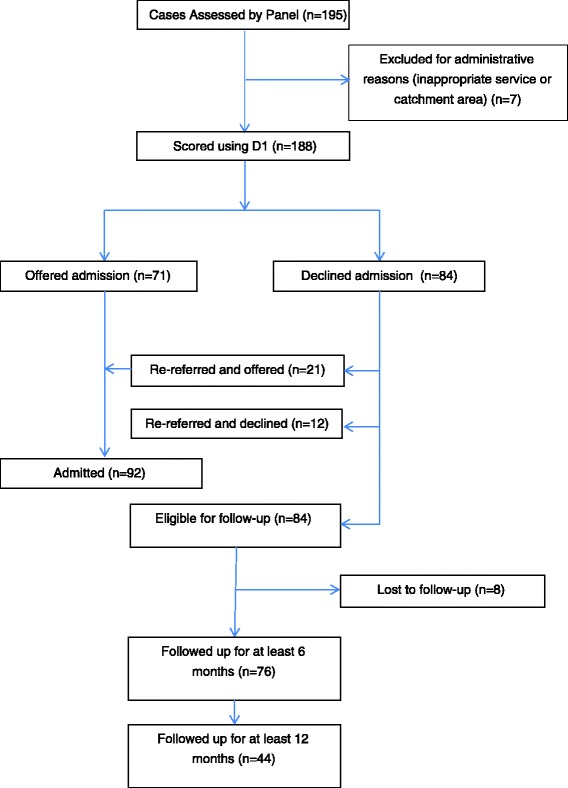
CONSORT Flow of cases considered for inclusion

Figure 3 shows the flow of cases into the referral system before and after consideration by the panel. Follow-up data was available at 6 months for 76 non-admitted patients (47 % of unique referrals, 90 % of unique non-admitted cases) and at 12 months for 44 patients (27 % of unique referrals, 52 % of unique non-admitted cases). Cases were lost to follow-up if they were discharged as patients under the Mental Health Act or could not be traced by the prison from where they were originally referred.

Of those non-admitted referrals followed up for 12 months (*n =* 44), six (14 % of non-admitted patients) had committed offences during that time period. This is comparable to general reconviction rates for successfully discharged MSS patients of 15 % over two years found by Maden et al. [19] and 47 % over 6 years reported by Coid et al. [20] and does not suggest that this population was at additional risk of criminality as a result of the decision not to admit. D1 score appeared to be predictive of offending post-referral, but did not reach significance at six months (AUC = .74, 95 % CI 0.58–0.91, *p =* 0.156), possibly due to low statistical power, or at 12 months (AUC = .58, 95 % CI 0.35–0.81, *p =* .411).

## Discussion

In this study, we sought to examine the predictive accuracy of the DUNDRUM triage measures when used to structure professional decision-making by a panel of psychiatrists and managers for a UK forensic MSS. We found that, for a population sample of adult forensic patients with severe mental illness, the D1 triage measure was significantly predictive of the decision to admit a patient, and could successfully classify 68 % of cases, more if making strict comparisons between levels of security. We also found that panel decisions made in this way differed significantly from clinical judgement of suitability alone made without the DUNDRUM toolkit.

Despite the strong overall internal consistency of the scale, not all items within the D1 triage measure were individually predictive of admission nor consistent with a single scale. Consistent with previous findings [11], the two items relating to, respectively, history of and future risk of self-harm did not load positively on the scale and did not significantly predict admission. This may be due to a mismatch between these items – which may apply to any psychiatric inpatient—and what might be considered the ‘latent’ structure of the D1 questionnaire as a guide to the relative need for therapeutic security of individuals with a history of violent or other serious offending. Whilst an individual’s self-harm is certainly a consideration in admission to psychiatric services, it would not be a defining concern in offering admission to a forensic bed in the same way as, for example, a long offending history or the need for restrictions on the patient’s access to a telephone. Previous research by Gray et al. has shown that violence risk assessments do not predict self-harm, and vice versa, suggesting that the self-harm items of the D1 are measuring an important clinical construct, but not one related to admission to forensic services. However, removal of the self-harm items did not result in a significantly improved predictive accuracy for the measure, and more recent work [16] has shown that some forensic assessments, including the HCR-20 and SAPROF, are predictive of both violence and self-harming behaviour.

Within this sample, two further items were also not individually predictive of admission: an item relating to Institutional Behaviour (S10); and another relating to Complex Risk of Violence (S9), which was significant only before correction for multiple testing. The first finding is somewhat easier to understand within this sample, which featured a high proportion (33 %) of referrals from low secure or open wards whose primary presentation was aggression and/or behavioural disorder, but not a forensic history. As is the case with the items relating to self-harm, individuals likely to score relatively highly on this item would not necessarily be considered suitable for admission to a forensic service due to a low risk of serious violent or other re-offending. The second item, relating to Complex Risk of Violence, would seem to be an important driver of admission but did not reach the same level of significance as a predictor. A possible explanation for this, which we will consider further below, relates to personality disorder: the wording of the item awards high scores to cases where there is evidence of severe personality disorder (3/4) or psychopathy (4/4).

Those who are violent in the absence of active mental illness, or who are violent due to a combination of mental illness and personality disorder are often more difficult to treat and are more likely to need specialist forensic mental health treatment programmes. One would expect this item to be positively predictive of admission to a secure forensic hospital, as indeed it has been found to be by Flynn, O’Neill, McInerney, et al. [11] Serious offenders with PD were not considered by the admissions panel in this study because in the UK, serious offenders with PD have for some time been part of a separate but parallel forensic system, either as part of the former Dangerous and Severe Personality Disorder (DSPD) Programme, or the new Offender PD Pathway [21]. Thus, the Complex Risk for Violence item would be associated with admission in Ireland and other countries, such as the Netherlands, with a single forensic system; but not in the UK where separate pathways operate for SMI and PD.

Despite the significant differences between clinician recommendation and panel decision, it was encouraging that, of those referrals who were not offered admission on the basis of their D1 scores, only a small proportion—comparable to that of ‘treated’ forensic MSS patients—subsequently went on to reoffend. Although this is not the only outcome of interest to forensic services, and ideally self-injury, mortality and recall would also have been considered in the study, it does suggest that the panel’s use of the D1 measure improved the transparency, accountability and speed of the admissions procedure, and significantly reduced waiting lists, without introducing any additional risk to the public.

### Limitations

There are a number of limitations of this study that should be considered. Firstly, since the DUNDRUM measures were used ‘naturalistically’ to support decision-making by the admissions panel rather than administered independently by a researcher, there is a risk of confirmation bias in that decisions made together with the tool may be self-affirming. The panel may have scored the tool to fit with a particular decision rather than made an objective decision based on the D1 rating, or, conversely, there may have been a ‘Halo effect’ of the process of rating the D1 measure that then influenced the decision in a particular direction. Whilst this is, of course, entirely acceptable and even—to an extent—desirable from the perspective of clinical practice, it does limit the generalizability of the findings as to the validity of the D1 since the predictor and outcome variables were not independent.

Second, the follow-up study was limited to an interview with clinical teams to identify offending, and would not have had the same accuracy as a review of criminal records conducted via, in the UK, the Police National Computer (PNC). Specifically, the true rate of offending may have been under-reported as clinicians may not have been aware of all convictions. There was also a high rate of loss to follow-up (*n =* 33, 39 %) due to loss of contact with services, which again may have influenced the accuracy of the reported offending rate.

## Conclusions

Use of a structured professional judgement measure, the DUNDRUM-1 and 2, assisted a forensic service in the UK to prioritise admissions and make transparent, evidence-based decisions about whether to admit and the urgency of that admission. The assessments also showed good internal and predictive validity and were significantly different to those made by individual assessing clinicians.

Forensic services have an ethical duty to patients, the public and general adult services to assess patients swiftly and to provide a response to referrals that is proportionate to the level of security adequate to the patient’s needs as well as the protection of the public, and to do this in an accountable fashion [22]. Extending the use of SPJ from use by individual clinicians to group-level decision-making, supported by clinical managers, is an encouraging potential method for implementing these goals.

## Abbreviations

AUC: Area Under the (receiver-operant characteristic) Curve
D1: DUNDRUM-1 Triage Security Measure
D2: DUNDRUM-2 Triage Urgency Measure
FLS: Forensic Low Security
HSH: High Security Hospital
MSRAG: Medium Secure Recidivism Assessment Guide
MSS: Medium Secure Services
MSU: Medium Secure Unit
PD: Personality Disorder
PICU: Psychiatric Intensive Care Unit
PNC: Police National Computer
ROC: Receiver-Operant Characteristic
SAPROF: Structured Assessment of PROtective Factors
SMI: Severe Mental Illness
SPJ: Structured Professional Judgement
